# Variable Magnification With Kirkpatrick-Baez Optics for Synchrotron X-Ray Microscopy

**DOI:** 10.6028/jres.111.018

**Published:** 2006-06-01

**Authors:** Terrence Jach, Alex S. Bakulin, Stephen M. Durbin, Joseph Pedulla, Albert Macrander

**Affiliations:** National Institute of Standards and Technology, Gaithersburg, MD 20899; Department of Physics, Purdue University, West Lafayette, IN 47907; J. Pedulla Associates, Silver Spring, MD 20906; Advanced Photon Source, Argonne National Laboratory, Argonne, IL 60439

**Keywords:** diffraction limit, Kirkpatrick-Baez, microscopy, multilayer mirror, x-ray optics

## Abstract

We describe the distinction between the operation of a short focal length x-ray microscope forming a real image with a laboratory source (convergent illumination) and with a highly collimated intense beam from a synchrotron light source (Köhler illumination). We demonstrate the distinction with a Kirkpatrick-Baez microscope consisting of short focal length multilayer mirrors operating at an energy of 8 keV. In addition to realizing improvements in the resolution of the optics, the synchrotron radiation microscope is not limited to the usual single magnification at a fixed image plane. Higher magnification images are produced by projection in the limit of geometrical optics with a collimated beam. However, in distinction to the common method of placing the sample behind the optical source of a diverging beam, we describe the situation in which the sample is located in the collimated beam before the optical element. The ultimate limits of this magnification result from diffraction by the specimen and are determined by the sample position relative to the focal point of the optic. We present criteria by which the diffraction is minimized.

## 1. Introduction

In 1948 Kirkpatrick and Baez [1] provided one of the first practical solutions to the problem of optical imaging with x-rays, overcoming the absence of traditional refractive lenses by showing that a pair of cylindrical mirrors can provide the point-to-point focusing required for image formation. Kirkpatrick-Baez (KB) optics have since been employed primarily for the imaging of radiation from plasmas [2–4]. The enormous advances in source brightness at x-ray synchrotrons have stimulated new interest in various forms of x-ray microscopy and fluorescence microanalysis using KB optics with multilayer-coated mirrors in demagnification to provide spots as small as a few micrometers which are rastered across samples to give images [5]. While zone plates offer higher resolution, KB optics are valued for their larger collecting areas.

While the microprobe is limited to collecting only one pixel at a time, a true imaging microscope collects all of the pixels in parallel and could in principle be much faster. We have constructed a KB microscope of short focal length using multilayer mirrors, and this instrument has already shown promise as a fast imaging system using a laboratory x-ray source [6]. We have now applied it to recording absorption images of thin specimens in hard x-ray synchrotron beams with the goal of enhancing image acquisition times for the study of dynamical systems. We report here the significant optical difference between operation with a conventional divergent source (critical illumination) and a highly collimated synchrotron radiation beam (Köhler illumination), the improvements in resolution that we have realized with synchrotron radiation, and the achievement of magnifications greater than expected with KB optics.

These are achieved by the transition from a real image into a geometric projective image. Projective x-ray imaging is normally performed by placing the sample in a diverging beam after an optical element (Fig. 3c) rather than in the collimated beam before an optical element (Fig. 3d). We describe the consequences of our unconventional geometry on the projective magnification and the limits to that magnification imposed by diffraction.

## 2. Experimental Details

**Fig. 1:**
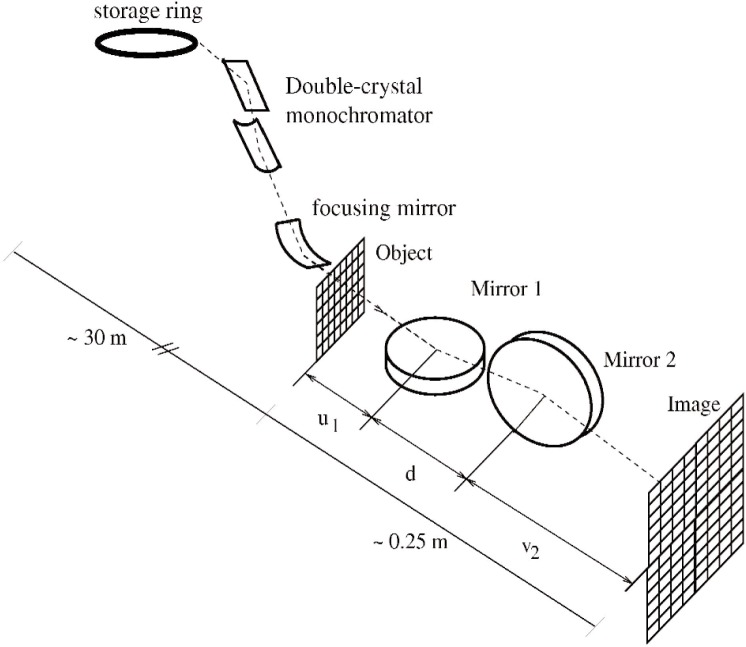
Kirkpatrick-Baez configuration at the Advanced Photon Source experimental station 1-BM-C: X rays from the Advanced Photon Source storage ring are first monochromatized by a sagitally-focusing double-crystal monochromator, and then further focused vertically by a curved mirror. Despite the double focusing, the divergence of the x-ray beam striking the object is several orders of magnitude smaller than what is practical with typical laboratory sources. The focal lengths of the two mirrors and the inter-mirror separation *d* are selected for the same distance from the object to the image.

Our basic arrangement for KB microscopy with synchrotron radiation is illustrated in Fig. 1, where the mirrors are actually Bragg reflectors consisting of carefully fabricated carbon-platinum multilayer coatings. Kirkpatrick and Baez showed that the two curved mirrors each obey the standard equation, with the image plane determined by the focal lengths of the mirrors and the object distance in the usual way

$$\frac{1}{u}+\frac{1}{v}=\frac{1}{f},$$ (1)

where *u* and *v* are the object and image distances from a mirror with radius of curvature *R* and angle of incidence *θ* = *θ*_B_, whose tangential focal length is simply *f* = (*R* sin *θ*_B_)/2. When this “lens maker’s law” is applied separately to the two mutually perpendicular mirrors, the combined equations which constrain a fixed object to have the same image plane for both mirrors are known as the Coddington equations [7]:

$$\frac{1}{u_{1}}+\frac{1}{v_{1}}=\frac{1}{f_{1}},$$ (2a)

$$\frac{1}{u_{2}}+\frac{1}{v_{2}}=\frac{1}{f_{2}}.$$ (2b)

The parameters *u_i_* and *v_i_* are the respective object and image distances for the two mirrors, whose centers are separated by *d*, i.e., *u*_2_ = *u*_1_ + *d*, and *v*_2_ = *v*_1_ − *d* (see Fig. 1). By analogy, the KB optics can be approximated as a simple lens, except that the lens has different focal lengths for the two transverse directions. The requirement of a simultaneous focus for both directions constrains the system to a single set of values *u_i_*, *v_i_* for a real image. The image magnification is simply *v_i_*/*u_i_*, so the magnifications are also different in the two transverse directions. We reduced the extreme astigmatism by using mirrors of different curvatures. Our previous study of the imaging properties of this microscope, using a standard laboratory x-ray source, showed that the images were fully consistent with these equations [6].

The multilayer coatings on the two mirrors were previously characterized with x-ray reflectometry, surface figure, and roughness measurements, and proved to be of exceptional quality. Starting with superpolished, precision-grade glass mirror blanks, fifty 2 nm C/1.2 nm Pt bilayers were sputter-deposited. The first-order Bragg reflection used in the KB microscope had a measured reflectivity of over 80 %, consistent with an interfacial roughness of less than 0.34 nm. High reflectivity and sharp interfaces are essential for good throughput and low background levels in the microscope images. At an energy of 8.05 keV, the experimentally determined focal lengths of the mirrors are *f*_1_ = 27 mm and *f*_2_ = 48 mm, respectively. The configuration was analyzed at this energy with the SHADOW ray-tracing program [8], for a distance between mirrors of *d* = 45 mm. The theoretical resolution simulated, 200 nm, is consistent with the resolution limits of spherical KB optics estimated by Prince [9].

After fully characterizing the prototype KB microscope with sealed-anode x-ray sources in the lab, this instrument was tested at the Advanced Photon Source, using x-rays at an energy of 8 keV. Station 1-BM-3 provided doubly-focused x rays with a flux of over 10^12^ photons per second into an area less than 1 mm^2^. Despite the double focusing, the beam divergence was less than 10^−4^ radians, which is effectively a parallel beam when compared to laboratory sources.

## 3. Results

**Fig. 2:**
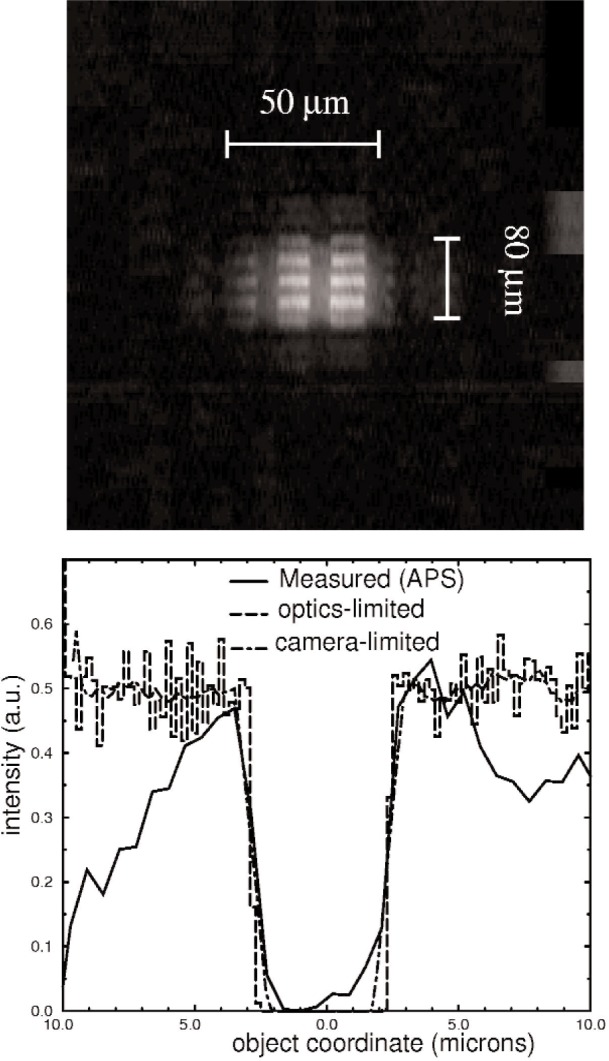
Image obtained with the Kirkpatrick-Baez microscope: Upper photograph shows the magnified image of a 1500 line/inch Au grid recorded with an x-ray CCD camera. Total exposure time is 30 ms. The square grid appears rectangular due to the different transverse magnifications of the microscope. Bottom plot shows the data from the image for the shadow of one 5 µm wire in the grid (solid line), indicating the change from maximum to minimum intensity occurs in about 1 µm. The dashed line denoted “optics limited” is the result of a ray-tracing calculation, which indicates an ideal resolution limit of about 200 nm. The “camera limited” line convolutes the ideal curve with the finite pixel size of the CCD camera (8 µm), showing good agreement with the data.

A sample image is shown in Fig. 2. It consists of the magnified image of a 1500 lines-per-inch Au wire grid located at a distance *u*_1_ = 3.1 cm, which was obtained using an x-ray CCD camera located at a distance *v*_2_ = 17.2 cm. The observed horizontal and vertical magnifications were 10 and 3.6, respectively. The magnifications expected from the first and second mirrors at the correct distance for focus *v*_2_ = 11.9 cm would be 5.4 and 1.6 respectively.

**Fig. 3:**
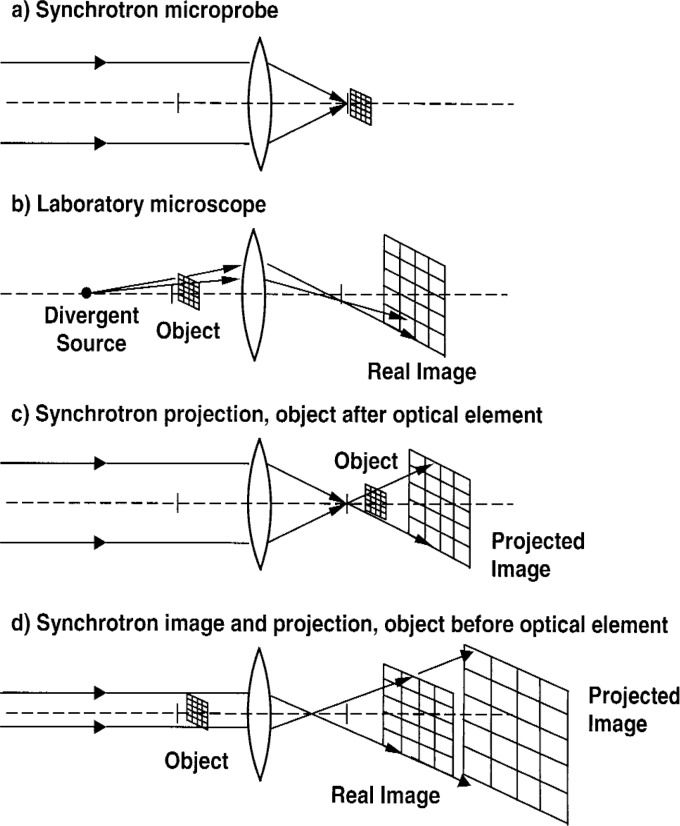
Lens model for a synchrotron KB microprobe, a laboratory KB microscope, conventional projection using a synchrotron light source, and the synchrotron KB microscope in the limit of geometrical optics: a) The microprobe focuses the nearly parallel x rays from the synchrotron down to the smallest possible spot at the focal point. This spot is then rastered across the specimen, while the transmitted intensity or a secondary yield is recorded and an image is formed, one pixel at a time. b) The laboratory microscope collects the rays from the object plane, where the object is typically back-illuminated by a diverging x-ray source, and focuses the image onto the image plane. The entire real image is formed at the same time, instead of one pixel at a time. c) Standard projection imaging with a synchrotron source uses an optical element to create a small diverging source behind which the object is located. d) In the case described here, the sample illumination is limited to a bundle of highly parallel rays through the object, located before the optical element (KB mirrors). A real image is observed behind the element, but the depth of focus is effectively very large (“projected image”).

Our acquisition time for the grid sample was 30 ms, nearly a thousand times faster than images acquired with the lab source. If we use the observed resolution to define a pixel area of 1 µm^2^, then the observed field of view (50 µm × 80 µm) corresponds to 4000 pixels. If this image were obtained one pixel at a time, as with a microprobe (Fig. 3a), at least 4 s would be required at the current best acquisition time of 1 ms/pixel [10].

Figure 2 also shows a line scan across a single wire of the grid (solid line), revealing a 1 µm width to the observed resolution of the Au wires. Also shown on the same plot is the ideal resolution of the wire expected for the optics by ray tracing, represented by the dashed line (“optics limited”). In the simulation nearly the entire edge drop occurs in one computational step, corresponding to an object resolution of only 200 nm. To better compare the simulation to the data, the computed curve was averaged over a length corresponding to the effective 8 µm pixel size of the x-ray camera divided by the horizontal magnification. This is plotted as the dot-dashed curve (labeled “camera-limited”), and more closely matches the profile of the data.

The observed 1 µm resolution of the data could be due to intrinsic optical aberrations, as well as camera resolution. The same image obtained with the lab source [6] had a resolution of no better than 4 µm (see Fig. 3b). Simulations also indicate that the smaller beam divergence at the synchrotron leads to improved resolution because less of the mirror surface is illuminated, making it less sensitive to figure error and other imperfections.

The observation that good images were recorded at distances *v* > *v_i_* with higher magnification than the real image caused us to reexamine the optical principles of the absorption microscope using synchrotron radiation, irrespective of the constraints implied by the Coddington equations Eqs. (2a,b). The usual premise when imaging with a lens is that the object is a source of diverging rays (except when the object distance is infinite). To an excellent approximation, however, we can treat the incident synchrotron radiation as a collimated beam. If we assumed only geometrical optics and that the specimen is a purely absorbing object, then there would be no divergence in the x rays transmitted by the specimen.

In a highly parallel incident beam, the specimen modifies the spatial intensity distribution, and the lens transfers this distribution onto the diverging outgoing rays. The resulting optical behavior is illustrated in Fig. 3d. The lens directs the parallel incident rays onto the focal spot a distance *f* from the lens. Assuming perfect collimation, the modification of the incident beam intensity profile is independent of the object position—the depth of focus is effectively very large.

This means that a projection image is collected downstream of the lens independent of the object position. Furthermore, there is no single image plane for a given object position: an image, which is the spatial modification of the intensity due to absorption by the sample, would be observed at *any* position downstream of the optic (assuming zero incident beam divergence). For *v* > *f_i_* the magnification increases linearly with distance from the focal spot:

$$M_{i}=\frac{v-f_{i}}{f_{i}}.$$ (3)

The variable magnification has been tested by an extensive set of images obtained with the synchrotron x-ray beam. Of course, for *v* = *v_i_*, *u* = *u_i_*, it is still true that *M_i_* = *v_i_*/*u_i_*.

There are several observations concerning the use of this instrument in the geometric optics limit. First, instead of being limited to a fixed magnification, greater magnifications would be found by simply moving the camera further from the microscope (Fig. 3d). This would reduce the need for higher resolution detectors: a 1 µm feature could be magnified until it is several times larger than the 8 µm pixel size in the camera, for example. Second, if geometrical optics were the only consideration, the ultimate resolving power would be determined largely by the synchrotron divergence and figure error in the mirror substrates, which we have already determined from simulations to yield a theoretical resolution as small as 200 nm. Finally, the sample position should not be very sensitive to the correct distance *u*_1_.

Diffraction effects constrain the above considerations of KB optics in the projective mode. Illumination of an object with a plane wave is in fact the starting point of the Abbe theory of image formation [11], in which a coherent plane wave diffracts from a specimen that is treated as a set of diffraction gratings. The Abbe theory further describes how these diffracted plane waves are focused by the lens onto its back focal plane, where the intensity distribution forms a Fourier transform of the object, and each point on the focal plane is a source of spherical waves which interfere to form an image at the image plane. We consider the limits of resolution due to both the optical elements and the object itself.

**Fig. 4:**
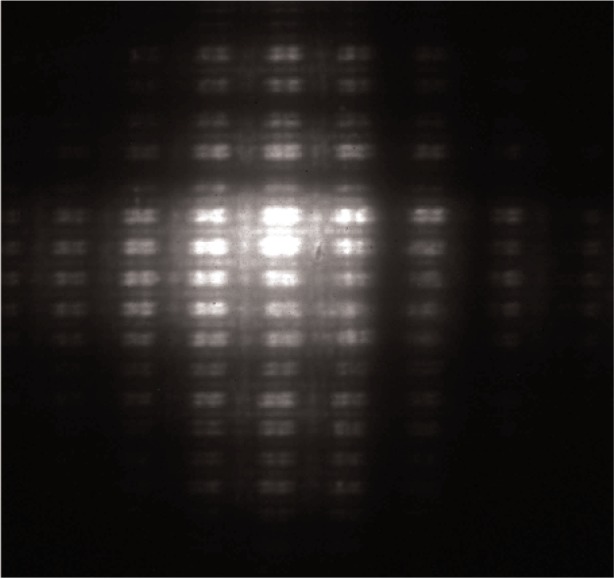
Image obtained with the Kirkpatrick-Baez microscope under conditions of projection where Fresnel diffraction is apparent. For *u*_1_ = 41 cm, *v*_2_ = 41 cm, large diffraction effects are visible in the images of the grid wires and between the grid wires.

Figure 4 shows an image of the grid in which diffraction effects clearly limit the quality of the image. The image was taken with the wire grid at a distance *u*_1_ = 41 cm and the camera at a distance *v*_2_ = 41 cm. The image was obtained with the microscope at the Advanced Photon Source on the 1-ID beamline, which has a beam divergence even smaller than that of the 1-BM beamline. The aperture of the first mirror (beam intercepted at the angle of the first Bragg condition at *θ* = 1.4°) is 0.65 mm. At a wavelength of *λ* = 0.154 nm, the Rayleigh limit of the optics is 7.6 nm, far smaller than our observable resolution limit.

If we now consider Fresnel diffraction from the wires in the grid, the deflection of the first interference maximum from the edge of the image is given by [12]

$$\Delta y={\left(u_{1}\lambda \right)}^{1/2}.$$ (4)

Using the values above gives a deflection of 7.9 µm, which is consistent with the observed diffraction. We can thus set limits to the practical magnification of projection images. For an object at *f_i_* < *u_i_* < 2*f_i_*, we assume that the diffracted rays are intercepted by the KB optic and refocused to an image at *v_i_*. This is no longer the case in the projection mode, where *v* > *v_i_*. In that case, the divergence angle Δϕ of a ray diffracted by the object is converted by the optic into a divergence angle

$$\Delta \phi^{\prime }=\frac{u_{i}}{v_{i}}\Delta \phi$$ (5)

at the image, where from (3), $\Delta \phi \approx {\left(\lambda /u_{1}\right)}^{1/2}$ for first-order Fresnel diffraction. The deflection *∆y′* of the diffracted ray at the image, which is 0 at *v* = *v_i_*, will be given generally by

$$\Delta y^{\prime }=\left(v-v_{i}\right)\Delta \phi^{\prime }.$$ (6)

The deflection of the diffracted beam for *v* > *v_i_* is thus

Δy′=(v−vi)vi(uiλ)12, (7)

and the ratio of the magnified deflection of the first order diffracted ray to the magnified image is

Δy′y′=(v−vi)fi(uiλ)12(v−fi)viy. (8)

We apply the above calculation to our KB optics for the geometry of the image in Fig. 2. For the object at *u*_1_ = 3.1 cm, we can tolerate ∆*y*′ = 8 µm, which is the limit of our camera resolution. This leads to a maximum distance of the camera of *v* = 79 cm without the perception of diffraction effects, at which point the horizontal magnification of the image is 28. At this point the magnified image of a wire in the grid would be 140 µm wide. Because of the short focal lengths of the mirrors, it is evident that small changes in specimen position *u*_1_ result not only in changes in the magnification of images in the projection regime but also in the degree to which the projected images are limited by diffraction. Unlike the assumption of geometrical optics, diffraction effects do depend on the position of the specimen in front of the optic for a perfectly collimated beam. In particular, Eq. (5) indicates that setting up the optics for a larger real image magnification (large *v_i_*/*u_i_*) permits a larger magnification in projection before the effects of diffraction become visible. By way of comparison, images taken at a specimen position *u*_1_ = 4.1 cm instead of *u*_1_ = 3.1 cm, for example, should show visible diffraction effects at the camera position of Fig. 2, where they were not previously visible.

The projection images discussed so far consider only the transmitted beam, but similar images can be obtained from beams diffracted from a crystalline sample as well. Dynamical diffraction from nearly perfect crystals would essentially preserve the synchrotron divergence allowing for the use of the KB microscope to obtain variable magnification in x-ray topography. It is also possible for diffraction from a given specimen to produce both parallel and diverging rays.

Let us consider the example of a silicon wafer with a patterned overlayer of lattice-mismatched Ge on the surface. Diffraction of an incident synchrotron x-ray beam from the substrate will produce a perfectly reflected beam of parallel rays, while diffraction from the distorted regions at the edge of Ge features could show noticeable divergence. The KB optics could be configured as a synchrotron microscope to image the perfectly reflected beam to show an absorption map of the Ge features, or it could be configured as a real image microscope and directly focus the rays from the distorted regions, producing a surface distortion map.

In summary, we have demonstrated the considerations that make the operation of a short focal length KB microscope in a highly collimated synchrotron radiation beam different from operation with a conventional divergent source. Tests at a low-divergence synchrotron beamline of a KB x-ray microscope produced test images with resolution of 1 µm or better, fields of view of 50 µm or more, and image acquisition times as low as 30 ms. Because of the unique aspects of a low-divergence beam, images in projection are obtained beyond the focus for a real image and the magnification in the projection mode varies with the distance of the camera beyond the focal point. Unlike simple geometrical optics, the effect of diffraction by the sample on the image depends on the focal length of the optics and the position of the sample. We have provided criteria that show the limits of projection magnification with KB optics and the effect of sample position.
